# The Effects of Testosterone on the Brain of Transgender Men

**DOI:** 10.1089/andro.2021.0008

**Published:** 2021-12-23

**Authors:** Leire Zubiaurre-Elorza, Sebastian Cerdán, Carme Uribe, Carmen Pérez-Laso, Alberto Marcos, Ma Cruz Rodríguez del Cerro, Rosa Fernandez, Eduardo Pásaro, Antonio Guillamon

**Affiliations:** ^1^ Department of Methods and Experimental Psychology, Faculty of Psychology and Education, University of Deusto, Bilbao, Spain.; ^2^ Instituto de Investigaciones Biomédicas Alberto Sols, Consejo Superior de Investigaciones Científicas, Madrid, Spain.; ^3^ Institute of Neuroscience, University of Barcelona, Barcelona, Spain.; ^4^ Departamento de Psicobiología, Universidad Nacional de Educación a Distancia, Madrid, Spain.; ^5^ Departamento de Psicología, Facultade de Ciencias da Educación, Universidade da Coruña, A Coruña, Spain.

**Keywords:** transgender men, testosterone, astrocytes, glutamine, androgenic anabolic steroids, MRI

## Abstract

Transgender men (TM) experience an incongruence between the female sex assigned when they were born and their self-perceived male identity. Some TM seek for a gender affirming hormone treatment (GAHT) to induce a somatic transition from female to male through continuous administration of testosterone. GAHT seems to be relatively safe. However, testosterone produces structural changes in the brain as detected by quantitative magnetic resonance imaging. Mainly, it induces an increase in cortical volume and thickness and subcortical structural volume probably due to the anabolic effects. Animal models, specifically developed to test the anabolic hypothesis, suggest that testosterone and estradiol, its aromatized metabolite, participate in the control of astrocyte water trafficking, thereby controlling brain volume.

## Introduction

Gender identity is one's sense of being a male or a female. The American Psychological Association^1^ defines it as “a person's deeply-felt, inherent sense of being a boy, man, or male; a girl, woman, or female; or an alternative gender (e.g., genderqueer, gender nonconforming, gender neutral) that may or may not correspond to a person's sex assigned at birth or to a person's primary or secondary sex characteristics.” Transgender men (TM) are persons assigned as female at birth who, however, during childhood, peripuberty, or later in life permanently feel they are male and experience gender incongruence/gender dysphoria. They desire a social transition from female to male and, in some cases, but not all, look for a somatic transition by means of a continuous treatment with testosterone. The European prevalence of TM is 2.6 in 100,000 individuals.^2^

When gender incongruence emerges prepuberally and, depending on the protocol used by the endocrinologist, the transgender boy could receive puberty blockers at the beginning of puberty^3^ to stop the growth of secondary sexual characteristics and the discomfort they could cause. This treatment gives the transgender boy time to reach the legal age to decide for himself on receiving continuous testosterone treatment and, perhaps, surgical sex reassignment.

Some TM seek a gender affirming hormone treatment (GAHT) to induce a somatic transition from female to male through the continuous administration of testosterone. The goal of the trans masculine treatment is virilization, which induces the secondary sexual characteristics of a man, and the cessation of menses. TM are treated with doses typically used to treat hypogonadism. Testosterone administration routes include esters, gels, or patches to keep hormone levels within the male physiological range (300–1000 ng/dL).^4^ Since transdermal administration can result in somewhat lower testosterone levels, in those circumstances concomitant progestin administration is needed.^3^

Signs of masculinization appear after ∼3–6 months of treatment. The effects of the virilization treatment in TM have been reviewed. TM experience increased body and facial hair, decreased fat and increased lean mass, a deepening voice, cessation of menstruation, clitoral enlargement, increased sexual desire, and decreased gender dysphoria, anxiety, and depression.^5^

From a clinical perspective, it has been stated that the hormone treatment of TM seems acceptably safe over the short and medium term, but solid clinical data are lacking.^6^ The most common gender affirmation surgery in TM is masculine chest reconstruction. A minority undergo oophorectomy or hysterectomy. Less common is genital reconstruction by phalloplasty or metoidioplasty.^4^ Psychologically, hormone-treated TM report less social distress, anxiety, and depression.^7^

Testosterone, directly or through its reduced or aromatized metabolites, exerts multiple physiological functions, such as stimulating muscle mass and strength,^8^ regulating osteoclastic and osteoblastic activities in the bone,^9^ stimulating erythropoiesis,^10^ and fat distribution.^11^ Moreover, administered in hypogonadal males, testosterone improves sexual desire, depression, and quality of life.^12^

From a molecular-genetic perspective, androgen receptor (AR) and estrogen receptor (ER) polymorphisms have been associated with transgender women^13,14^ and estrogen receptor polymorphisms to TM.^15^ A recent study in a large and homogeneous sample of adult TM expressing gender incongruence from childhood onward found that the TM population is associated with polymorphisms of the estrogen receptors alpha [(ERα); *Xba*I-Erα, (rs9340799)] and/or estrogen receptor beta [(Erβ); (CA)n-Erβ, (rs113770630)].^16^ It has been hypothesized that subtle changes occurring during brain development underlie the TM brain and behavioral phenotypes.^17,18^ The few existing scientific studies that directly address the effects of pharmacological doses of sex steroids on the structure of the TM all note strong effects on the brain structure. Here we briefly address the brain structural characteristics in TM before and after the masculinization treatment. We also provide an explanation that has emerged from changes observed with a recently developed animal model.

## The Brain of TM Before the Masculinization Treatment

Brain structural quantitative magnetic resonance imaging (MRI) studies on macroscopic brain structure such as cortical and subcortical volume, cortical thickness, and white matter microstructure show that the brain of TM, before their gender affirming testosterone treatment, shows a mixture of feminine, masculine, and defeminized morphological traits.^17,19^

Several studies have approached the white and gray matter of TM before the masculinizing treatment. At the macroscopic level, volumetric analyses show that the intracranial volume of an adolescent TM is like that of cisgender females. However, regionally, volume decrements have been found in the left superior medial frontal cortex of TM when compared with that of cisgender girls and larger right cerebellum volumes compared with those of cisgender girls.^20^ Regarding subcortical structures, the volume of the putamen in TM is like that of cisgender males and larger than in cisgender females.^21^ With respect to surface area, cisgender males show a larger surface area than cisgender women in the superior temporal lobe and orbitofrontal regions, whereas cisgender males showed relatively higher brain volume and surface area than cisgender women.^22^ A recent study published by the ENIGMA Transgender Working Group^23^ points out that TM present lower volumetric values as well as less surface area than cisgender men.

Female and male cisgender subjects show sex differences in cortical thickness, with the four lobes showing a thicker cortex in females than in males.^22,24^ Cortical thickness does not differ statistically between TM and cisgender females. Nevertheless, in areas in which male and female cisgender do not differ, like parietal and temporal regions, TM show a thicker cortex than cisgender males. However, unlike cisgender females, TM did not differ from cisgender males in the prefrontal orbital region.^21^ This gives them their cortical thickness phenotype.^17^

With respect to the white matter microstructure, it should be remembered that male and female cisgenders differ.^25^ Males show greater fractional anisotropy values than cisgender females.^22,26^ We confirmed greater fractional anisotropy values in cisgender men than cisgender women. TM, like cisgender men, have greater anisotropy values than cisgender women in the right superior longitudinal fasciculus and the forceps minor, but TM differ from both cisgenders with respect to the corticospinal tract. Consequently, we suggested developmental sex differences between the three groups.^27^ Moreover, widespread significant differences were reported in mean diffusivity between groups in almost all white matter tracts. Mean diffusivity describes the magnitude of water diffusion within brain tissue. Cisgender women had the highest mean diffusivities, followed by TM with the next highest.^28^ Investigating the structural connectome, it was found that lobar interhemispheric connective was lower in TM than in transgender women or the male and female cisgenders.^29^

## The Brain of TM Under Testosterone Treatment

The effects of GAHT on the brain have recently been reviewed in both transgender men and women.^30^ In this study, we focus primarily on longitudinal studies investigating the effects of testosterone on gray matter and white matter structure in TM. This is because the explanations provided in the existing literature on the effects of GAHT on the brain come from brain structural studies in TM^31^ and an animal model using adult female rat androgenization.^32^

With respect to gray matter, the first study, in a sample of only six TM under testosterone treatment, reported an increase in total brain volume and the hypothalamus.^33^ Later, using a longitudinal design, increased cortical volume was found in the cortex and the right thalamus in TM under at least 6-month virilizing treatment with no effect on the ventricular system. In addition, a thicker cortex was also reported in the bilateral postcentral gyrus and unilaterally in the inferior parietal, lingual, pericalcarine, and supramarginal regions of the left hemisphere, and the cuneus and middle frontal region of the right hemisphere. Moreover, serum testosterone changes positively correlated with large clusters of cortical thickness in the lateral occipital, inferior parietal, and fusiform areas. Similarly, a positive correlation was found between the free testosterone index and cortical thickness in the occipital region, inferior and superior parietal, and fusiform areas.^31^ Cortical thickening has been confirmed in several regions^34,35^ as well as increased volume in subcortical structures.^36^

Regarding white matter microstructure, increases in fractional anisotropy values were first reported in the right superior longitudinal fasciculus and the right corticospinal tract.^27^ Interestingly, hierarchical regression analyses showed that increments in fractional anisotropy could be predicted by the prehormonal treatment testosterone index.^37^ Similar results have been reported regarding increases in the left cingulum and decreased mean diffusivity in several corticocortical tracts.^30^ Increases in fractional anisotropy were also reported in the fronto-occipital tract.^34^ Thus, testosterone treatment in TM increases morphological brain parameters of both gray and white matter.

## Testosterone Exerts an Anabolic and Anticatabolic Effect in Brain Tissue of TM Under Treatment

Testosterone androgenic-virilizing and anabolic effects cannot be teased apart. TM feel they are men before receiving testosterone treatment and it should be underscored that their gender feelings are well established before the hormone affirming treatment. The sole objective of the treatment is to virilize their body. Consequently, the effects of testosterone treatment on brain morphology do not affect their firm gender identity as men but relieve their gender incongruence as they meet their bodily gender expectations. Testosterone has effects on those regions in which ARs are expressed and this should focus our attention on ARs and ERs in brain tissue.

Up to the present time, we do not know if the increases in anisotropy, volume and cortical thickness values are mediated through the ARs or the ERs. We cannot discard the participation of either or both kinds of receptors. Cortical and subcortical structures express estrogen^38,39^ as well as ARs.^40^ Aromatase^41,42^ and reductase^43^ activities have been detected in the human brain. Testosterone can be aromatized to estradiol, and dihydrotestosterone reduced to 5α-androstane, 3β, 17β-diol that also binds to ERs, particularly ERβ.^44^ Therefore, both possibilities remain open. Sex steroids can act through genomic and nongenomic pathways,^45,46^ and ARs and ERs can act in a ligand-independent manner.^47,48^ Animal studies suggest that testosterone upregulates AR.^49^ Thus, we face a myriad of possibilities when a female body receives supraphysiological doses of testosterone to be virilized.

The continuous exposure of TM to pharmacological doses of testosterone might exceed the usual metabolic mechanisms of androgens and their metabolites in the brain. Testosterone and its metabolites stimulate protein synthesis. In men, supraphysiological doses of testosterone, combined with strength training, increase fat-free mass and muscle size and strength^50^ as well as increase protein synthesis and net muscle protein balance.^51^ In sports, androgens induce performance enhancement in women.^52^ In TM, fractional anisotropy increases might reflect a greater richness in axonal microtubules and macromolecules. Consequently, we proposed a process in brain cells that would be like the anabolic one described in the muscle.^31^

An anticatabolic effect has also been hypothesized to explain testosterone effects on muscle. Androgens could exert anticatabolic actions by interfering with glucocorticoid receptor expression.^53^ This would induce a positive nitrogen balance. Glucocorticoid receptors have been identified in the human brain.^54^ If testosterone influences brain cells as it does muscles, this hypothesis should be taken into consideration.^31^

## Testosterone Affects Brain Morphology Changing Astrocyte Size in Adult Female Rats

The fact that testosterone is administered to TM is not the only reason justifying the development of an animal model to study the effects of testosterone on brain tissue. Indeed, testosterone has also been prescribed to treat hypogonadism as well as psychosexual and erectile dysfunctions and fatigue in men.^55–57^ Moreover, the anabolic androgenic steroids (AASs), which are synthetic substances derived from testosterone, are widely used by bodybuilders and weightlifters. Although the hormone might be neuroprotective,^58^ chronic administration of AASs is associated with psychiatric disorders in men and women,^59–62^ and a neurodegenerative potential has been suggested.^63^

**FIG. 1.**
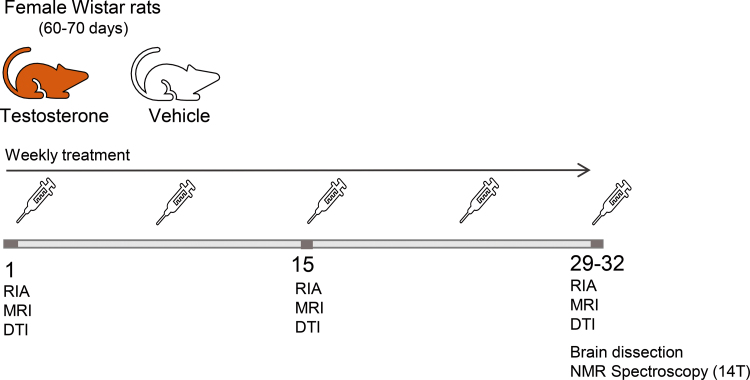
Experimental design of a longitudinal study to androgenize adult female rats. Animals were weekly injected with testosterone or vehicle. Every 15 days, blood from the tail was collected for immunoassay and MRI was acquired. At the end of the experiment, animals were euthanized and brain was dissected to obtain the metabolic spectrum. DTI, diffusion tensor imaging; MRI, magnetic resonance imaging; NMR, nuclear magnetic resonance; RIA, radioimmunoassay.

We developed an animal model to test the anabolic hypothesis on the cortical effects of testosterone.^32^ We designed a longitudinal study using adult 80-day-old female rats injected weekly with a testosterone dose equivalent to that administered to TM. The anabolic hypothesis predicted increases in fractional anisotropy values, because of increased protein synthesis in brain cells. A response to maintain osmotic homeostasis by the brain cells would be observed as changes in cortical volume. Cortical volume and anisotropy values were measured *in vivo* using T2- and diffusion-weighted images, respectively. Proton magnetic resonance spectroscopy (^1^H MRS) was used *ex vivo* to assess the metabolic profile of neurochemical metabolites in various brain regions (Fig. 1).

**FIG. 2.**
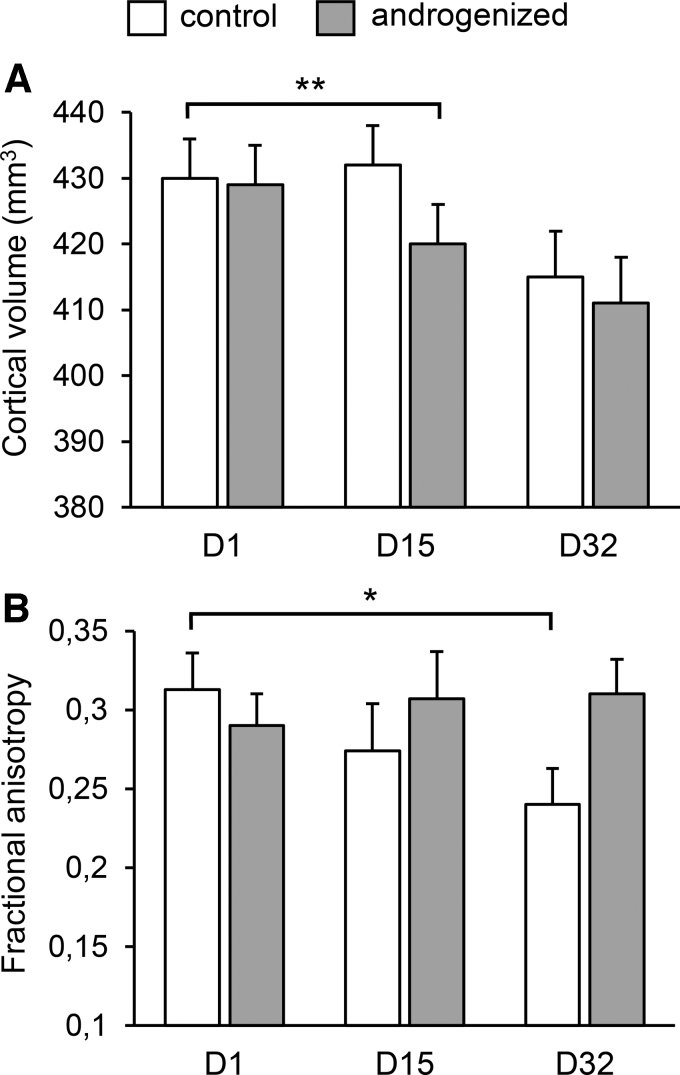
Effects of testosterone on the cortical volume **(A)** and fractional anisotropy **(B)** in adult androgenized female rats. Androgenized rats showed a sharper decrease in cortical volume while maintaining fractional anisotropy values against/despite aging effect seen in control animals. Data from Perez-Laso et al.^39^ *p < 0.05; **p < 0.01. FA, fractional anisotropy.

The main hypothesis was verified—although fractional anisotropy values decreased steadily in control female rats over the course of the experiment, no change in their values was observed in androgenized rats (Fig. 2). Decreases in control females could be explained by aging, as is seen in humans.^64–66^ This suggests that testosterone prevented age-dependent decreases in fractional anisotropy.

Both control and androgenized females showed a decrease in cortical volume that was more pronounced in androgenized females (Fig. 2). Rats achieved their largest cortical volume at 2 months of age, with volume declining from that age^67^; that decline was sharper in androgenized females by day 15 of treatment, and the metabolite profile was also different.

The relative concentrations of some metabolites that function as osmolytes showed different fates in androgenized females than in their controls. Adult androgenized females showed a decrease in *myo*-Inositol (mI), glutamine (Gln), and glycine + Gln (Gly+Gln). Linear regression analyses indicated that these decreases were due to the increases in testosterone levels. Changes in mI and Gln significantly affected the *N*-acetyl-aspartate/mI ratio, the aspartate/Gln ratio, and the γ-aminobutyric acid (GABA)/aspartate ratio. Since mI and Gln behave as major osmolytes, testosterone levels may alter volume regulation processes, and this may underlie the changes observed in cortical volume. This suggests that the administration of supraphysiological doses of testosterone affects water content in brain cells. It seems that testosterone, or its metabolites, are involved in the osmotic homeostasis of the cell.

mI, as well as being a major osmolyte involved in the regulation of cell volume under osmotic stress conditions,^68^ is also an astrocyte marker and the precursor of the phosphatidylinositol second messenger system.^69^ In our design,^32^ the relative concentration of mI was negatively correlated with the levels of testosterone and associated with a shrinkage of brain cells. The mI- and Gln-driven intracellular water efflux decreased astrocyte volume and hence cortical volume.

**FIG. 3.**
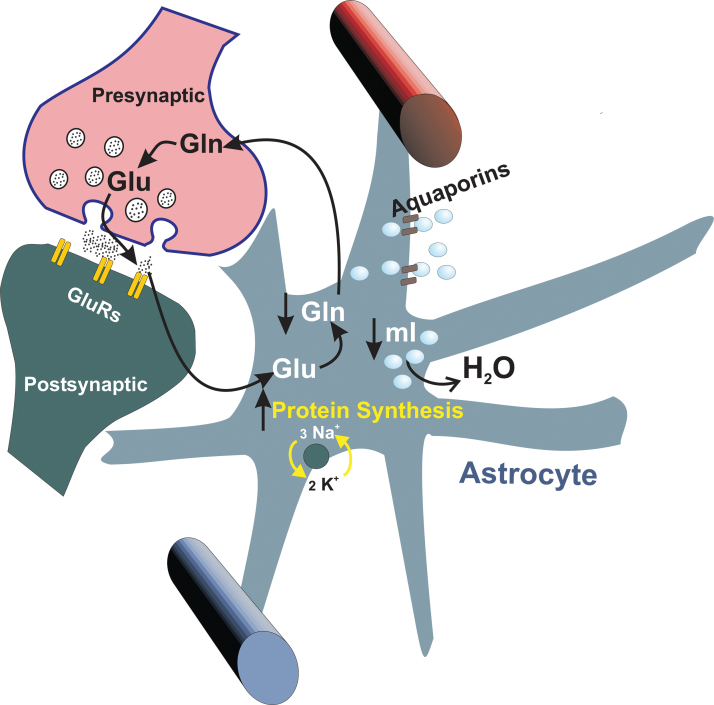
Suggested mechanism to explain cortical volume decrease in subjects under supraphysiological doses of testosterone. Increased protein synthesis attracts water into the astrocytes. In turn, to maintain the osmotic equilibrium, astrocytes efflux water to the extracellular space by means of osmolytes such as mI and Gln whose relative content decreases. Decreases in the Gln concentration might affect the Gln–Glu cycle. Gln, glutamine; Glu, glutamate; GluRs, glutamate receptors; mI, myo-Inositol.

In summary, testosterone increases molecular synthesis in astrocytes, inducing increased water intake, which increases cortical volume. To prevent astrocytes from swelling during the hormone treatment and maintain cortical volume constant, it was suggested that mI and Gln would be driven out from the astrocytes into the extracellular space and eliminated through the vascular system. This suggestion is supported by the observation that mI and Gln decreased their relative concentrations in androgenized adult female rats with respect to their controls (Fig. 3).

Decreased Gln levels might have other kinds of consequences. Astrocytes are the only cell type able to synthetize Gln, and Gln is the precursor of inhibitory (γ-amino butyric acid) and excitatory (glutamate [Glu], aspartate) neurotransmitters. When Gln is decreased because of its osmolyte function,^70^ the inhibitory and excitatory equilibrium might be affected. This was indicated because the ratios of GABA/aspartate and aspartate/Gln were also decreased.

Decreases in the astrocyte level of Gln should focus our attention on neuron–astrocyte relationships.^71^ A decrease in Gln may affect the Gln–Glu cycle.

## Concluding Remarks

The animal model already summarized suggests that supraphysiological doses of testosterone exert effects on the brain volume of adult female rats through the astrocytes and, to preserve osmotic homeostasis, the astrocytes then decrease osmolytes such as Gln and mI. In turn, decreases in Gln would affect the equilibrium of excitatory and inhibitory neurotransmitters (Fig. 3).

What can we learn from the animal model with respect to TM and AAS consumers? The structural brain MRI data we know come from TM during short-medium administration (around a year or less),^31,72^ whereas data from AAS consumers are from long-term consumption.^73,74^ Over 1-year's consumption of AASs by weightlifters is associated with a thinner cortex and smaller volumes of total gray matter, cerebral cortex, and putamen,^74^ but right amygdala enlargement and higher Gln/Glu ratio have also been reported.^73^ These reports suggest that the effects of testosterone and other AASs on brain structure depend upon the duration of the administration of these substances.

We do not know whether the effects seen in the brain of TM, weightlifters, and rats are produced by testosterone itself or by its reduced and aromatized metabolites or by all these hormones. In adult male rats, daily estradiol administration produced a decrease in cortical volume associated with increases in the relative concentration of Gln and other metabolites due to water depletion from astrocytes.^75^ The fact that both testosterone and estradiol drive a decrease in cortical volume in rats suggests that estradiol plays a role in the effect promoting an active mechanism of neurocellular water extrusion.

We should acknowledge the probable participation of other variables. Specifically, channel proteins such as Aquaporins that regulate water movements across neurocellular membranes.^76^ Aquaporin 4 (AQP-4) is located in the astrocytic end feet. This presence provides a link between microvascular blood flow and metabolic coupling between neurons and astrocytes. Testosterone upregulates AQP-4 expression in cultured astrocytes,^77^ and estradiol modifies AQP-4 expression. Thus, supporting the relationship between sex steroids and cerebral volume regulation.^78^ Moreover, AQP-4 downregulates Glu uptake in astrocytes.^79^ Therefore, AQP-4 becomes an excellent candidate for future research into the effect on the brain of the androgenization treatment of transgender men.

Finally, higher or supraphysiological doses of testosterone may increase the risk of psychiatric symptoms in persons with underlying hypomania, mania, or psychotic disorders.^80^ Changes in brain cortical volume are observed in both TM and weightlifters. Moreover, the animal model of the treatment indicates that water trafficking is affected in astrocytes and changes in the relative concentrations Gln and Glu are also observed. To contribute to the quality of life of TM, in light of these observations, we would suggest that an MRI scan be taken before receiving testosterone and every to 2 or 3 years during the routine follow-up of the treatment so as to safeguard quality of life.

## Abbreviations Used

AASs: anabolic androgenic steroids
AQP-4: Aquaporin 4
AR: androgen receptor
ER: estrogen receptor
ERα: estrogen receptors alpha
Erβ: estrogen receptor beta
GABA: γ-aminobutyric acid
GAHT: gender affirming hormone treatment
Gln: glutamine
Glu: glutamate
mI: *myo*-Inositol
MRI: magnetic resonance imaging
TM: transgeder men
